# Supplementary material for: A Review of the Tear Film Biomarkers Used to Diagnose Sjogren’s Syndrome
Source: Int J Mol Sci. 2024 Sep 26;25(19):10380. doi: 10.3390/ijms251910380 (PMC11476667; doi:10.3390/ijms251910380)
Supplement: Supplementary file 1 [file ijms-25-10380-s001.zip › ijms-3146531-supplementary.pdf]

Supplementary:

Table S1: Key Characteristics and Limitations of the 22 Studies Included

Author:	Year:	Country:	Study Type:	Population:*	Diseases Covered:	Sample	Sample Collection:	Buffer Used	Extraction method	Limitation:
Hsiao et al.	2022	Taiwan	Cross-sectional, case-control Observational Study	36	non-SS-DED; pSS-DED; and sSS-DED	Tear	Automated pipette	Saline	Standardized eye-flush procedure with normal saline	The study's cohort size is relatively small, limiting the generalizability of the findings. Additional studies with larger patient cohorts are needed for broader applicability. Further, a deeper understanding of pathophysiological and biochemical pathways is needed.
Kannan et al.	2023	India	Review	56	DED, SS	Tear	Schirmer's strips, microcapillary tubes	Protein extraction buffer (Ammonium bicarbonate/triethylamm	Collected then immediately flash frozen at -80C until processing	Potential incomplete or biased literature, methodological differences, and heterogeneity of the populations of the various included studies

								onium bicarbonate) or phosphate buffered saline (PBS)		
Carracedo et al.	2016	Spain	Review	N/A	DED, SS (Also congenital aniridia)	Tear (Also aqueous humour, retina)	N/A	N/A	N/A	Potential incomplete or biased literature, methodological differences, and heterogeneity of the populations of the various included studies
Hohenstein-Blaul et al.	2013	Germany	Review	N/A	DED, SS (Also diabetic retinopathy, cancer)	Tear	N/A	N/A	N/A	The text highlights challenges of clinical validation and standardization of tear biomarkers for routine use, limitation translatability of the research to the clinic.

Akpek et al.	2020	USA	Case-control Observational Study	62	SS-dry eye; non-SS dry eye; Non-SS, no dry eye	Tear	Miccrocapillary tubes	Cytokine Assay Buffer (Merck Millipore, Millipore Iberica, Madrid, Spain)	Collected then diluted 1:10 and frozen at -80C until analysis	The study's cohort size is relatively small, limiting the generalizability of the findings. Additional studies with larger patient cohorts are needed for broader applicability. Further, the subjects in the control group did not have a specific workout for SS other than medical history, revie of systems, and ocular surface and tear film exams. This means that it's possible to have mis- or undiagnosed SS. Finally, MUC5AC levels were measured in tears using ELISA, meaning the sensitivity could potentially be higher.
Altman et al.	2023	USA	Review	599	DED, SS (Also herpes epithelial keratitis, vernal keratoconjunctivi	Tear	Capillary tube, micropipette, Schirmer's strips	Varying (Phosphate buffer salin	N/A	The limited research in this field makes it hard to establish a baseline miRNA profile in human tears. Further, the lack of a standardized protocol limits reproducibility of the study.

					tis, primary open angle glaucoma, diabetic macular edema, diabetic retinopathy, and Alzheimer's disease)			e (PBS) for one study included		
Berra et al.	2021	Argentina	Cross-sectional Observational Study	90	DED, SS, and meibomian gland dysfunction	Tear	Filter paper disc	Phosphate-buffered saline	Collected then stored at -20C until processed. Processed using Micrococcus lysodeikticus agar diffusion assay in Mueller Hinton agar.	The findings may lack generalizability due to the population only including female subjects. Further, the study's cohort size is relatively small, limiting the generalizability of the findings. Additional studies with larger patient cohorts are needed for broader applicability.

Levine et al.	2021	USA	Retrospective Study	128	DED, SS	Tear	Lateral flow immunoassay test strips	N/A	N/A	The findings may lack generalizability due to the population only including older male US veterans that were seeking eye care. The cross-sectional nature of the study also prevents a causal relationship between variables to be determined. Further, the dendritic cells were counted manually, leading to potential biases and variations in the count.
Hamm-Alvarez et al.	2014	USA	Cross-sectional Observational Study	278	DED, SS	Tear	Schirmer's strips	CTSS Reaction Buffer (Biovision, Milpitas, CA)	Collected then placed on ice and analyzed within 4 hours of collection. Alternatively stored at -20C and analyzed within 24 hours, then stored at -80C until	The findings may lack generalizability due to the population only including female subjects. Also, the cross-sectional nature of the study also prevents a causal relationship between variables to be determined. Overall, additional studies with larger and more diverse populations are needed to validate this study's findings.

									quantification.	
Ponzini et al.	2020	Italy	Meta-Analysis	1093	DED, SS (Also diabetic retinopathy)	Tear	Schirmer's strips, capillary tubes, polyester wick, micropipette	N/A	Gel electrophoresis, ELISA, other undisclosed methods (Protocols not disclosed)	There is high heterogeneity among the studies included due to the differing ample sizes and geographical study locations. There may also be publication bias as there was a low number of studies included. Finally, dry eye patients were diagnosed based on signs and symptoms, so some patient may have undiagnosed SS.
Aqrawi et al.	2018	Norway	Cross-sectional Observational Study	27	DED, pSS	Tear	Schirmer's strips (HAAG-STREIT, Essex, UK)	N/A	In-solution protein digestion, then LC-MS using Ultimate 3000 RSLCnano-UHPLC system connected to a Q Exactive mass spectromet	The study's cohort size is relatively small, limiting the generalizability of the findings. Additional studies with larger patient cohorts are needed for broader applicability.

									er (Thermo Fisher Scientific, Bremen, Germany)	
Beckman et al.	2017	USA	Review	N/A	SS	Tear	N/A	N/A	N/A	Potential incomplete or biased literature, methodological differences, and heterogeneity of the populations of the various included studies
Urbanski et al.	2021	France	Cohort Study	90	DED, pSS	Tear	Schirmer's strip	Methanol	Collected and stored at -80C, then transferred to precooled homogenization Precellys tubes (Bertin Technologies, Montigny-le-Bretonneux, France) filled with	The study's cohort size is relatively small, limiting the generalizability of the findings. Additional studies with larger patient cohorts are needed for broader applicability. The ocular tests used for pSS diagnosis also had high variability. Further, the study does not give long-term follow-up information about the patients, which may paint a more complete picture of the disease's progression and stability

								ceramic beads and cold methanol. Afterwards, put through two grinding cycles, centrifuged, and frozen at -80C until analysis.	of the biomarkers studied.
Aqrawi et al.	2019	Norway	Cross-sectional Observational Study	35	Non-SS sicca; pSS; and non-SS non-sicca	Tear	Schirmer's strips (HAAG-STREIT, Essex, UK)	Phosphate-buffered saline (PBS)	Collected and stored at -80C until analysis (LC-MS)	The study's cohort size is relatively small, limiting the generalizability of the findings. Additional studies with larger patient cohorts are needed for broader applicability.
Khimani et al.	2020	USA	Comparative Study	28	Control, SS aqueous tear deficient (ATD), and non-SS ATG	Tear	Schirmer's strips (Haag-Streit, Mason, OH)	N/A	Collected and stored at -80C until processing and analysis. Measured	The study's cohort size is relatively small, limiting the generalizability of the findings. Additional studies with larger patient cohorts are needed for broader applicability. The control

									using a micro BCA protein assay kit)Thermo Fisher Scientific, Waltham, MA)	and experimental groups have different age ranges, potentially having a confounding effect on the data. Further, the use of impression cytology is limited in real-world application, so patient inclusion may have potential bias.
Pur et al.	2023	Canada	Systematic Review	1058	DED, SS, keratoconus, meibomian gland dysfunction	Tear	N/A	N/A	N/A	There were concerns of poorly-defined inclusion criteria, lack of any detailed descriptions of study subjects and settings, unidentified confounding variables, and a lack of protocol to handle known confounding variables for the studies included. Further, the AI methodologies used to identify and analyze the studies were not fully described, making it difficult to confirm the validity of the results. Additionally, there was limited data with small

									sample sizes, lack of healthy controls, and variability with the protocol utilized for the less common biomarkers studied.
Brignole-Baudouin et al.	2017	France	Cross-sectional Observational Study	311	DED, SS, meibomian gland disease	Tear	Schirmer's strips	Phosphate-buffered saline (PBS)	Collected then stored at 4C before analysis, then subsequently processed and extracted.	The study's cohort size is relatively small, limiting the generalizability of the findings. Additional studies with larger patient cohorts are needed for broader applicability.
Aqrawi et al.	2017	Norway	Experimental Study	22	pSS	Tear	Schirmer's strips (HAAG-STREIT, Essex, UK)	Phosphate-buffered saline (PBS) (Gibco, pH 7.4, ThermoFi	Collected then stored at -80C until analysis. Analy´d with LC-MS	The study's cohort size is relatively small, limiting the generalizability of the findings. Additional studies with larger patient cohorts are needed for broader applicability.

								sher Scientific, Oslo, Norway)		
Karns et al.	2011	USA	Comparative Study	4	SS	Tear	Schirmer's strips	Phosphate-buffered saline (PBS)	Collected then frozen at -80C until processed. Processed by incuabation in protein Lobind microcentrifuge tube with PBS on a shaker overnight, then frozen at -80C until use.	Because the assay utilized targets lactoferrin, its applicability may not extent to other tear biomarkers. Further, the study's cohort size is relatively small, limiting the generalizability of the findings. Additional studies with larger patient cohorts are needed for broader applicability.
Shinzawa et al.	2018	Japan	Observational Case Series	17	DED, SS	Tear	Glass capillary tube	N/A	Collected the nseparated by centrifugation and stored in -	The study's cohort size is relatively small, limiting the generalizability of the findings. Additional studies with larger patient cohorts are

									80C until aanlysis.	needed for broader applicability.
Hargreaves et al.	2019	Switzerland	Case-control Observational Study	28	pSS	Tear	Schirmer's strips	N/A	Collected then placed on ice and analyzed within 4 hours of collection. Alternatively stored at -20C and analyzed within 24 hours, then stored at -80C until quantification.	No significant correlations between disease duration and CatS or CatL expression were discovered. This is possibly due to the low number of pSS patients enrolled in the study. Additionally, the study did not include patients with other rheumatic diseases and secondary Sjögrens or patients with non-immune sicca symptoms. As such, they cannot conclude that the findings are specific for primary Sjögren Syndrome.
Edman et al.	2018	USA	Cross-sectional Observational Study	156	DED, SS	Tear	Schirmer's strips, glass capillary tube	Phosphate-buffered saline (PBS)	Collected then frozen at -80C until processed	The study's cohort size is relatively small, limiting the generalizability of the findings. Additional studies with larger patient cohorts are needed for broader applicability.

*Patient population is based on the number of patients tears were collected from. It may be different from the total patient population of the study. i.e. Some patients only had saliva or serum collected from them
